# Understanding access to general practice through the lens of candidacy: a critical review of the literature

**DOI:** 10.3399/BJGP.2024.0033

**Published:** 2024-09-27

**Authors:** Carol Sinnott, Akbar Ansari, Evleen Price, Rebecca Fisher, Jake Beech, Hugh Alderwick, Mary Dixon-Woods

**Affiliations:** Health Foundation professor of healthcare improvement studies, The Healthcare Improvement Studies Institute, University of Cambridge, Cambridge.; Health Foundation professor of healthcare improvement studies, The Healthcare Improvement Studies Institute, University of Cambridge, Cambridge.; Health Foundation professor of healthcare improvement studies, The Healthcare Improvement Studies Institute, University of Cambridge, Cambridge.; The Health Foundation, London, UK.; The Health Foundation, London, UK.; The Health Foundation, London, UK.; Health Foundation professor of healthcare improvement studies, The Healthcare Improvement Studies Institute, University of Cambridge, Cambridge.

**Keywords:** access to care, candidacy, general practice, qualitative research

## Abstract

**Background:**

Dominant conceptualisations of access to health care are limited, framed in terms of speed and supply. The Candidacy Framework offers a more comprehensive approach, identifying diverse influences on how access is accomplished.

**Aim:**

To characterise how the Candidacy Framework can explain access to general practice — an increasingly fraught area of public debate and policy.

**Design and setting:**

Qualitative review guided by the principles of critical interpretive synthesis.

**Method:**

We conducted a literature review using an author-led approach, involving iterative analytically guided searches. Articles were eligible for inclusion if they related to the context of general practice, without geographical or time limitations. Key themes relating to access to general practice were extracted and synthesised using the Candidacy Framework.

**Results:**

A total of 229 articles were included in the final synthesis. The seven features identified in the original Candidacy Framework are highly salient to general practice. Using the lens of candidacy demonstrates that access to general practice is subject to multiple influences that are highly dynamic, contingent, and subject to constant negotiation. These influences are socioeconomically and institutionally patterned, creating risks to access for some groups. This analysis enables understanding of the barriers to access that may exist, even though general practice in the UK is free at the point of care, but also demonstrates that a Candidacy Framework specific to this setting is needed.

**Conclusion:**

The Candidacy Framework has considerable value as a way of understanding access to general practice, offering new insights for policy and practice. The original framework would benefit from further customisation for the distinctive setting of general practice.

## Introduction

Access to health care is an increasing focus of interest in health systems worldwide.^1^^–^^3^ However, policy-level conceptualisations of access have tended to be dominated by considerations of supply of appointments and by measures of utilisation.^4^ These conceptualisations obscure how people become and are processed through systems as ‘candidates’ for care. This happens in ways that are structurally, organisationally, institutionally, culturally, and professionally constructed, and that are strongly influenced by inequities as well as by the operating conditions of services. Narrow interpretations of access also risk obscuring differences between services and how access might differ between and across patient groups. The Candidacy Framework, originally developed to explain access to health care by vulnerable groups,^5^ seeks to address the need for a more comprehensive way of understanding access.

Describing how people’s eligibility for health care is a *‘continually negotiated property’*,^5^ at the heart of the Candidacy Framework is the recognition that access is a dynamic and contingent process subject to multiple diverse and interdependent influences. As originally proposed, the framework identifies seven overlapping features of candidacy (Box 1). The boundaries between the features are fluid, reflecting how eligibility for medical attention is constantly being defined and redefined through interactions between individuals, professionals, systems, and structural influences. Candidacy is, for example, affected by individuals and their socioeconomic contexts, how services seek to constitute and define appropriate healthcare attention and intervention, macro-level structures and allocation of resources, and the decisions and actions of those who provide care. The Candidacy Framework also recognises that access may require considerable work on the part of users, and that the complexity of that work may create socially patterned barriers to care.

**Box 1.** Summary of the seven features of the Candidacy Framework for understanding access^5^

**Identification**
How people recognise their symptoms as needing medical attention or intervention is important to how they assert a claim to candidacy.
**Navigation**
Using services requires knowledge of the available services and depends on having the practical resources to use them.
**Permeability**
The ease with which people can use services depends on how many and what kinds of criteria people have to meet to use them, and on cultural and other alignments between services and individuals.
**Appearances**
Appearing at services involves people making a claim to candidacy. It requires a set of competencies and sociocultural alignments.
**Adjudications**
Professional judgements about patients’ candidacy strongly influence individuals’ access to attention and interventions.
**Offers and resistance**
Offers of care may be made that may be accepted (utilisation) or refused (non-utilisation) by individuals.
**Operating conditions**
The perceived or actual availability and suitability of resources has a major impact on the local production of candidacy, as do other relevant operating conditions.

Candidacy has already proved useful in understanding influences on access in secondary mental health,^6^ palliative care,^7^ and maternity services.^8^ An especially important area for research on access is general practice, which has a key role in providing continuous and coordinated care, promoting health, and providing an entry point to other parts of the healthcare system.^9^^,^^10^ Current challenges, including declining numbers of GPs and increased patient need, have contributed to public dissatisfaction and renewed political interest in ‘fixing’ access to general practice.^3^^,^^11^^,^^12^ However, if the full range of influences on access are not addressed, then proposed solutions risk being misdirected, ineffective, or liable to amplifying inequities. An overarching characterisation of candidacy as it applies to general practice is therefore much needed. We aimed to address this need through a critical review of the literature.

**Table:** How this fits in

Dominant conceptualisations of access to health care are often framed in terms of speed and supply — these approach es risk obscuring important as pects of people’s experiences of access. The Candidacy Framework was developed to study access to health care by people in vulnerable groups. This study confirms the salience of the Candidacy Framework for under stand ing access in the setting of general practice, offering new insights for policy and practice.

## Method

We sought to conduct an author-led,^5^ iterative, analytically guided review to characterise the distinctive features of general practice that may be relevant to candidacy. We were guided by the principles of critical interpretive synthesis,^5^ particularly in its use of a critical orientation, use of theoretical sampling, and the creative, imaginative approach to synthesising a wide range of literature.^13^ Given our aim of determining how well the Candidacy Framework is suited to understanding access to general practice, a full critical interpretive synthesis was not appropriate, since critical interpretive synthesis is geared towards *de novo*development of theory and constructs.

We aimed to identify peer-reviewed research literature, opinion pieces, editorials, and grey literature relevant to access to general practice. Articles were only eligible for inclusion if they related to the context of general practice or particular patient cohorts in this setting (for example, those with diabetes). Although we did not limit our search by countries or years of publication, we did not, consistent with the critical interpretive synthesis approach, seek comprehensiveness. We preferentially sampled articles relevant to the UK, with a view to informing understanding and further research on access to general practice in this setting.

We initially used a broadly defined search strategy, including purposive selection of material likely or known to be relevant, and guided by Starfield’s foundational article describing key features of primary care.^9^ Starting from the articles that cited and were cited by this article, three reviewers sought to identify literature that described characteristics of general practice that might be relevant to understanding access. We searched electronic databases, internet search engines such as Google, websites of think tanks, and recent tables of contents for key journals relevant to general practice, including the *British Journal of General Practice*, *Family Practice*, and the *BMJ*. In some cases, searches revealed ‘pockets’ of literature on a discrete component of access or general practice. When this occurred, we conducted mini-scoping searches of the literature on that topic to identify the most relevant articles for our purpose. Concurrently with this review, all authors were also working on an extensive systematic search and screening of almost 5000 citations (summarised on this webpage: https://www.health.org.uk/sites/default/files/2024-03/Methods%20for%20summary%20options%20list%20FINAL.pdf) to catalogue previous attempts at improving access to general practice. More than 30 articles identified in this way were used as important sources for this review.

These searches were supplemented by an additional structured search to identify systematic reviews on access to primary care, using the PubMed search string ((systematic review[Title]) AND (access[Title])) AND ((general practice[Title/Abstract]) OR (primary care[Title/Abstract])), to ensure no key literature had been missed.

We engaged in constant reflexivity to inform the emerging theoretical interpretation of the data. Consistent with critical interpretive synthesis principles, searching for relevant articles proceeded alongside sampling, critiquing, and analysing included articles in a dynamic and mutually informative process, with emerging findings used to guide literature sampling to maximise relevance and theoretical contribution. We assessed that we had reached theoretical saturation when 10 consecutive new articles addressing different features of the framework were judged not to be modifying the findings.^14^^,^^15^

Articles were curated in NVivo (version 12). We did not conduct formal quality appraisal, since our primary interest was conceptual and our sources were highly heterogenous, so their contributions were assessed using subjective judgement rather than methodological criteria.^16^ Themes relating to access to general practice were extracted by three researchers. Given the aims of our review, we synthesised findings using a focused coding approach,^15^ to align themes extracted from the literature with the candidacy feature (Box 1) to which they most closely related. This component of the review was more closely aligned with the framework method of qualitative analysis than critical interpretive synthesis.^17^

## Results

We included 229 articles in the final synthesis:^4^^,^^9^^–^^11^^,^^18^^–^^242^ 173 academic articles, 26 editorials or opinion pieces, 15 think tank outputs, seven policy documents, and eight books, PhD dissertations, media publications, or government publications; 195 were from the UK, eight from North America, nine from other European countries, and one from Australia, while 16 articles used data from multiple countries(see Supplementary Box S1).

Our analysis found that the Candidacy Framework offers considerable value as a way of understanding access to general practice. Organising the literature using the features of Candidacy enables structured insights into wide-ranging influences on access, although our analysis also suggests that the framework would benefit from further customisation for the distinctive setting of general practice. A detailed summary of the literature relating to each of the seven features is provided in Supplementary Table S1, and each is briefly presented here.

### Identification of candidacy

The Candidacy Framework emphasises that whether and how people recognise their symptoms as needing medical attention is important to their asserting a claim to candidacy. Our analysis (see Feature 1, Supplementary Table S1 for details) confirmed the salience of *identifying candidacy* for understanding access to general practice. Although most people have symptoms of something most of the time,^18^ such as feeling tired/ run down, having joint pains, and so on,^19^ that could in principle make them candidates for general practice care, only a minority are in fact brought to attention. This ‘illness iceberg’^20^^–^^22^ makes the question of how people make judgements about their candidacy an important one.

Our analysis identified patients’ uncertainties about the boundaries of general practice and difficulties matching their symptoms with what they understand as the threshold for warranting attention at a practice. This perceived threshold is not fixed: capabilities for self-care, understandings, and fears vary significantly, to the extent that a symptom seen as trivial to one person might be fraught with significance to another.^23^^–^^25^ In assessing their candidacy, people appear to be influenced by multiple factors (see Feature 1, Supplementary Table S1 for details), many unrelated to the symptoms themselves,^26^^–^^35^ and often powerfully shaped by issues of illness identity.^36^ People with some conditions (such as cancer) may feel their claim to medical attention is relatively secure, even for symptoms unrelated to their cancer, but those with equally serious but perhaps less socially understood conditions (such as congestive cardiac failure), may feel less able to assert a claim of eligibility for care.^29^^,^^36^^–^^39^ Media campaigns appear to have a role both in encouraging people to assess their candidacy and in legitimating help-seeking for particular conditions or symptoms,^23^^,^^32^^,^^40^ but can negatively affect candidacy by creating the perception that certain thresholds must be reached before attendance at a *‘service in crisis’*^41^ can be justified.^35^^,^^42^

A distinctive characteristic of candidacy for general practice is that for some services, particularly preventive care (such as vaccinations or health checks), candidacy may be identified by the services and enacted through invitations, rather than patients identifying it themselves.^9^^,^^43^^–^^47^

### Navigation

Our analysis (see Feature 2, Supplementary Table S1 for details) confirms the relevance of the *navigation* feature, which describes people knowing what services are available and having the transport, social, occupational, and financial resources to use them. The literature points to the growing complexity of navigation in general practice. Recent institutional changes in the organisation of general practice are especially powerful in their effects on people’s ability to understand the available services and use them in a way deemed appropriate by the system.^48^^–^^53^ Some of these have led socially patterned challenges in navigability,^27^^,^^42^^,^^49^^,^^50^^,^^54^^–^^56^ as exemplified by widespread introduction of triage systems, including call-back telephone appointments or online triage tools.^57^^–^^59^ Triage has the potential to ease navigation for patients by directing care to the service or professional deemed most appropriate by the triager, but it may impact on people’s agency to navigate to the service of their choice in their own way.^60^^–^^62^ Although digital triage facilitates flexibility in how primary care is requested, patients’ experience of requesting access to care through digital channels is mixed.^27^^,^^59^

Navigation is also impacted by the recent rapid diversification of skill mix in general practice.^39^ People may now be offered an appointment with a diverse range of professionals — such as a pharmacist, social prescriber, physiotherapist, or physician associate — rather than necessarily with a GP.^63^^–^^65^ Role diversification, which is intended to provide patients with a fuller range of services and potentially release GPs’ time, may complicate navigation and introduce challenges for continuity of care — for example, by making more complex the matching of patients to the most appropriate professional and reassuring patients of the equivalency of care provided by different practitioners, and by creating the potential for duplication and inefficiencies.^9^^,^^39^^,^^49^^,^^54^^,^^64^^,^^66^ Although skill mix has been associated with increased access to appointments, it appears that it only improves experiences of access if patients are happy with, trust, and have confidence in the choice of practitioners they are offered.^64^ Patients also appear to face difficulties accessing the same practitioner for follow-up appointments in new access systems, with knock-on effects for their acceptance of the newer roles.^67^

The relative importance of speed of appointment, time of appointment, or continuity of care varies by patients’ age, morbidity, and other factors (see Feature 2, Supplementary Table S1 for details) and may add further to complexity of navigation.^4^^,^^68^^–^^71^ Challenges in navigation in general practice may mean that people instead attend what they perceive as more straightforwardly navigable options, such as emergency departments.^45^^,^^49^^,^^72^^–^^81^ Conversely, some may attend general practice when more appropriate services exist, such as direct-access physiotherapy.^50^^,^^69^^,^^74^^,^^82^^–^^86^ Attempts to support navigation in general practice are not uniform in their effects, in part due to the social patterning of health literacy: over half of the leaflets on systems navigation have been shown to be too complex for at least 15% of the UK population, and >80% of practice websites do not meet recommended ease-of-reading levels.^87^^–^^90^ In addition, patients’ needs for access vary depending on whether it is a one-off need that is transactional in nature or part of a long-term therapeutic relationship (for example, in the context of a chronic illness).^68^^,^^91^

### Permeability

*Permeability*refers to the ease with which people can use services: more permeable services do not, for example, require qualifications of candidacy (like referrals), extensive personal resources, or cultural alignment between themselves and their users. Our analysis suggests that general practice, traditionally a porous service by design, may have become more closed in recent years.

One impact on permeability of general practice is the growing diversity of options for requesting and receiving care. Remote care, for example, may reduce the physical effort required to attend face-to-face appointments.^4^^,^^92^^,^^93^ However, some changes intended to improve access in fact impair it — for example, by making appointments systems more opaque,^85^ and requiring patients to align with and have the capabilities needed to use such systems.^90^ Some changes in how appointments are booked require people to have specific resources to use them (such as a telephone with credit, the ability to answer call-backs at the time called, or the language capabilities to discuss needs remotely).^62^^,^^94^ Similarly, while online interactions have been welcomed by some patients, their use has made access more difficult for others, particularly those experiencing digital poverty or lacking in digital ability.^27^^,^^50^^,^^54^^,^^85^^,^^95^^,^^96^ Further, online consultations based on text may offer less scope to understand patient context, especially if patients feel uncomfortable sharing sensitive information via these tools.^97^^–^^99^

Receptionists play a key role in general practice permeability, since they often operate as gatekeepers to appointments and, increasingly, in directing patients towards particular types of professional.^64^ Their decisions and appointment allocations may be influenced by their sense of responsibility to practice colleagues,^53^^,^^72^ by practice culture on the value of speed of access versus continuity of care,^100^^,^^101^ or by patient reluctance to provide clinical information to them (as non-clinicians).^39^^,^^52^^,^^68^^,^^102^

### Appearances

*Appearances* at health services involves people asserting a claim to candidacy for medical attention, care, or interventions. Our analysis indicates that, in general practice, appearances are significantly influenced by patients’ ability to express their health needs during consultations. Patients may be sensitive to the pressured context in which healthcare encounters take place, experience uncertainty about the legitimacy of their symptoms, or have language difficulties,^94^^,^^103^ which constrain them from representing their true needs or ensuring they are heard.^23^^,^^104^^–^^107^ Although ‘one issue per appointment’ policies exist in some practices,^108^ multiple problems are discussed in most general practice consultations,^109^^–^^113^ making active management of consultations by healthcare professionals, for example, through agenda setting and ascertaining clear accounts of patients’ worries and reasons for attending, essential for ensuring that patients’ needs are met.^49^^,^^91^^,^^108^^,^^114^^–^^119^

Continuity of care affects appearances by facilitating patients to present as legitimate users, to ask questions, and to be involved in decision making.^69^^,^^120^^,^^121^ Without continuity, patients may feel that they must go through the process of presentation of self and presentation of need at every encounter.^106^^,^^122^ The form of appointment (face-to-face or remote) also impacts on the quality of patients’ appearances.^27^^,^^60^^,^^91^^,^^96^^,^^123^^,^^124^ Remote care, for example, may have negative consequences for shared decision making, promote more paternalistic medicine, and, in situations of digital poverty, increase inequalities.^27^^,^^95^^,^^96^^,^^125^

Although longer consultations potentially improve patients’ ability to assert their candidacy and are associated with improved patient outcomes,^106^^,^^115^^,^^126^^–^^129^ consultations in general practice remain tightly time-bound.^4^^,^^98^^,^^116^^,^^129^^–^^132^ Trials of interventions to increase consultation length show mixed results, perhaps indicating that consultation length functions primarily as a marker for other dimensions of quality of care.^115^^,^^127^^,^^130^^,^^133^^–^^137^

### Adjudications

Once someone has made an appearance at a service, the Candidacy Framework suggests that professional judgements (‘adjudications’) are made about their eligibility for different forms of care — for example, interventions like prescriptions or referrals, or, as is often the case in general practice, simply a dialogue between professional and patient.

Our analysis suggests that *adjudications*in general practice cover a very wide and diverse range of presenting symptoms and draw on professionals’ repertoires of routine judgements, typifications, and mind-lines (collectively reinforced, internalised, tacit guidelines).^138^ Further, these adjudications occur in the ‘inherently uncertain environment’ generated by the frequently undifferentiated early-stage problems that present to general practice.^139^^,^^140^ Adjudications appear to be influenced by factors as varied as patients’ age, sex, education, socioeconomic status, previous adherence to treatment, health behaviours, values, expectations, family, culture, and quality of life.^9^^,^^44^^,^^118^^,^^120^^,^^141^^–^^143^

Accordingly, adjudications draw on generalist expertise, including, where available, contextual longitudinal knowledge of the patient over time.^66^^,^^118^^,^^139^^,^^144^ Adjudications are also made in the context of the GP’s role as a gatekeeper for other parts of the healthcare system. This gatekeeping role (see Feature 5, Supplementary Table S1 for details), which is not present in all healthcare systems, is made more complicated by the expanding range of available services to which GPs can refer, with sometimes limited guidance on how these services should be used;^145^^,^^146^ by patients’ dissatisfaction with limited access to specialist care;^147^ and by the ability of some patients to use private specialist services, effectively bypassing the GP.^148^

Our analysis identified that a distinctive feature of access to general practice care is that many consultations may have a relational character that mean they are *‘not simply an exchange of facts, diagnoses, and prescriptions’*but can have intrinsic *‘therapeutic value, especially when embedded within an enduring relationship.’*^149^ (See also other references.^9^^,^^91^^,^^117^^,^^132^^,^^139^^,^^144^^,^^150^^–^^159^) Consequently, some adjudications in general practice may be oriented towards the therapeutic relationship being sustained, or empowering, enabling, or reassuring patients,^107^^,^^160^^–^^163^ even when the patient and the GP are not in a dyadic relationship of continuity.^161^

### Offers and resistance

Adjudications about individuals’ candidacy can result in ‘offers’ for active management including referrals, prescriptions, investigations, or advice, or approaches including watch and wait. These offers may be accepted or declined (‘resisted’) by patients. Our analysis suggests that how offers are made and responded to falls on a spectrum from paternalism (doctor-determined) to patient-led. Although patients might wish to lead decisions in one consultation but defer to GPs’ recommendations in another,^153^^,^^164^^–^^169^ approaches to decision making in general practice are generally *‘shaped over time through exposure to and reflection on a range of encounters’*^31^ and can occur over *‘a series of consultations’*.^169^

Our analysis further suggests that while the terms ‘offers’ and ‘resistance’ used in the original account of candidacy potentially imply that a refusal is somehow ‘negative’, refusals that incorporate patients’ values and preferences may represent exercise of choice and agency, and thus are examples of good care.^170^ However, some resistance may arise from misalignments in the negotiation of candidacy, since GPs and patients approach decision making using different perspectives and epistemologies. Accordingly, misunderstandings, lack of knowledge, and time constraints^43^^,^^78^^–^^80^^,^^171^ on the part of either party may all influence what offers are made and which are accepted. Significant work may be required of GPs to prevent misunderstandings and missed opportunities arising in the gulf between *‘the scientific “voice of medicine” and the experiential “voice of the*[patient’s] *lifeworld”’*.^172^ (See also other references.^34^^,^^139^^,^^164^^,^^173^^–^^175^) Other barriers to shared decision making include poorly designed decision tools, the time required, and patients’ health literacy.^176^^–^^179^

### Operating conditions

Our analysis of the literature strongly emphasises how candidacy for general practice care is influenced by what are termed ‘operating conditions’ in the Candidacy Framework, including local pressures and policy imperatives. Relevant operating conditions at national levels include GP contracts and policy initiatives on changes in care delivery, technological changes, such as new IT systems, and socioeconomic inequalities. An example of a policy with far-reaching consequences for candidacy is the Quality and Outcomes Framework. It incentivised access for patients with index conditions and led to reduced variation in care quality for these conditions, but was also associated with adverse effects on access for non-incentivised symptoms or conditions.^45^^,^^46^^,^^180^ Similarly, general practice funding formulas impact on candidacy through their effects on distribution of GPs. Lower funding and fewer GPs per head of need-adjusted population in deprived areas than affluent areas may result in knock-on problems for practices in deprived areas, such as unsustainably high workloads, and challenges of recruitment and retention of practice staff, with attendant threats to access.^4^^,^^50^^,^^54^^,^^66^^,^^91^^,^^152^^,^^181^^–^^184^

## Discussion

### Summary

Our critical review of a large literature has affirmed the overall salience of the Candidacy Framework in understanding access to general practice. Using the framework draws explicit attention to how access to general practice is not simply a matter of supply or speed of appointments, but is also a function of how people perceive their symptoms, identify general practice services as being able to meet them, have the resources (cognitive, physical, and others) to find their way to them, and can present their needs in a way that can be adjudicated on and subsequently processed, all in complex and resource-constrained environments. Although access to general practice in the UK is free at the point of care, barriers nonetheless exist, and may be especially consequential for some groups, especially those who are more disadvantaged. In emphasising that candidacy is highly dynamic, contingent, and subject to constant negotiation, the lens of candidacy allows access to general practice to be understood as an interplay between multiple actions, decisions, and forces, many of them socioeconomically and institutionally patterned. Our analysis also emphasises how understanding access to general practice simply in terms of a series of unrelated transactions or actionable requests (the ‘supply-focused’ model of access) is misleading and unhelpful.

Each of the seven features characterised in the original Candidacy Framework are relevant for general practice, but we also found that the framework requires additional customisation for the specific setting of general practice, with particular attention to the nature of relationships in general (involving repeat players), the increasingly diverse ways in which contact with general practice occurs and the implications for permeability and navigation, the trade-offs people may make in speed versus continuity and how these may vary depending on reasons for help-seeking (from one-off requests to long-term chronic illness management and therapeutic relationships), the increasingly variable ways in which patients may make ‘appearances’, and the constraints on any adjudications and offers given resource and capacity limitations (especially in specialist services).

As an example, we identified the need for conceptualisations of candidacy for general practice to recognise the highly recursive nature of the long-term relationships people may have with their practice,^238^ even when individual encounters may take place outside the idealised doctor–patient longitudinal dyad.^66^ This is important because patients’ experiences and learning from one care episode to the next accumulate over time, such that single episodes in general practice may be influenced by, and go on to influence, many aspects of candidacy. A further example of how the original Candidacy Framework needs to be adjusted for general practice is in its conceptualisation of offers and resistance and the role of relational continuity. Our analysis suggests that, while some requests for access are transaction-focused, some general practice patients may, instead of a specific intervention or ‘offer’, be seeking an interaction with a person that they know and trust.^161^^,^^162^ This makes relational continuity — which involves an ongoing therapeutic relationship between a patient and a clinician so that they ‘know each other well’ — a key consideration in thinking about access,^66^ and particularly in thinking about what good looks like for access. Our findings align with others^243^ in suggesting that processes for delivering relational continuity may need renewed attention for a general practice that is increasingly characterised by declining numbers of GPs,^63^ and fragmented and/or remote approaches to care.

### Strengths and limitations

We used an author-led approach in recognition that traditional systematic review methods were not suited to the goals of our review, and that a conceptual review of the type we attempted requires different criteria of comprehensiveness.^13^ Accordingly, our search and analysis was highly iterative: rather than aiming to exhaustively represent all relevant literature in general practice, we have offered here a theoretically grounded account based on what we have judged to be appropriate selections of material. The dependability of our results is supported by the multidisciplinary nature of our team and the range of methods we used to identify and select sources. It is possible, however, that our analytically guided searches missed some relevant literature. The implications of our choice to focus on conceptual contribution rather than formal appraisal of methodological quality, while consistent with other approaches,^244^ is difficult to assess.

Our study does not fully account for the increasing diversification of skill mix in general practice, given that the literature is still catching up with policy and practice-driven changes. At present, limitations of the literature mean, for example, that it is not clear whether patients perceive that seeing professionals other than GPs meets their needs for access. It is, however, likely that changes in skill mix may have impacts on continuity that are difficult to predict based on current evidence.^64^^,^^67^ A candidacy-informed approach is likely to support thoughtful changes to professional training, role development, and practice operations.^66^^,^^96^

### Comparison with existing literature

Policy-level approaches to understanding access have been dominated by considerations of supply and assessed using measures such as the number of GPs per head of population or proportion of people seen within 48 hours of requesting an appointment. Recent approaches have sought to go beyond this simplistic characterisation of access. Boyle *et al*,^4^ for example, describe three features in their account: physical access, timely access, and choice. A more detailed approach is that of Levesque *et al*,^245^ which conceptualises five dimensions of accessibility (approachability, acceptability, availability and accommodation, affordability, and appropriateness) and five corresponding abilities of populations (ability to perceive, seek, reach, pay, and engage). Access has also been framed as the ‘human fit’ between the needs and abilities of the population and the capacity and abilities of the healthcare workforce.^239^ For practitioners and policymakers, the relative strengths of the Candidacy Framework include its patient-centred approach spanning the entire patient journey, the emphasis given to characteristics of the healthcare encounter, and its value in recognising the needs of vulnerable groups and the powerful influences of socioeconomic and institutional conditions.^5^

### Implications for practice

Our analysis indicates that using the Candidacy Framework may help those working in general practice, practice organisation, or policy to think innovatively and comprehensively about where improvements to access are most needed, understand why previous efforts have failed, and identify promising solutions. For example, done well, gatekeeping of secondary care services by primary care is associated with better quality of care, more appropriate use of hospitals, and lower healthcare expenditure,^246^ but it does require general practice access.^147^^,^^148^^,^^240^ That in turn requires a well-informed understanding of the complex and diverse influences on access, starting with patient identification of candidacy through navigation, permeability, appearances, and adjudications, offers and resistance, and the contexts of operating conditions.

Similarly, continuity of care needs policy-level recognition as an important component of access to general practice.^66^^,^^241^^,^^242^ Because the supply-focused model has dominated policy responses, continuity has not been ‘designed in’ at policy level in the same way that fast access to appointments has been.^4^ Indeed, policy efforts to improve speed of access can have negative effects on continuity.^54^^,^^66^^,^^91^^,^^152^^,^^184^ New evidence indicating the positive associations between continuity and lower healthcare utilisation, morbidity, and mortality for all patients, regardless of pre-existing conditions, age, or frequency of contact,^238^ means future policy on access should strive to strike a balance between speed of appointment and continuity. Further customisation of the Candidacy Framework for the diversity of needs in general practice may help to support this.

In re-envisioning access to general practice through the lens of candidacy, our analysis further highlights how the boundaries between different elements of the framework are becoming more blurred. For instance, the shift to digital and remote care, and the widespread introduction of triage blurs the distinction between navigation, permeability, and appearances as the methods used to manage access (such as digital) are also increasingly used to respond directly to patients’ questions or requests.^4^^,^^27^^,^^96^ The growing complexity and overlaps between navigation, reduced permeability, and appearances may create new socioeconomically patterned barriers and potentially impact on adjudications and offers of care. At the same time, new ways of interacting with patients, such as through text-based online consultations, may be implicated in a re-imagining of the very nature of access. These developments not only require attention to what access means and what good looks like, but also to assessing system outcomes.
